# PolySialic Acid Nanoparticles Actuate Complement-Factor-H-Mediated Inhibition of the Alternative Complement Pathway: A Safer Potential Therapy for Age-Related Macular Degeneration

**DOI:** 10.3390/ph17040517

**Published:** 2024-04-17

**Authors:** Sheri L. Peterson, Anitha Krishnan, Diyan Patel, Ali Khanehzar, Amit Lad, Jutamas Shaughnessy, Sanjay Ram, David Callanan, Derek Kunimoto, Mohamed A. Genead, Michael J. Tolentino

**Affiliations:** 1 Aviceda Therapeutics Inc., Cambridge, MA 02142, USA; akrishnan@avicedarx.com (A.K.); alad@avicedarx.com (A.L.); dcallanan@avicedarx.com (D.C.); dkunimoto@avicedarx.com (D.K.); mgenead@avicedarx.com (M.A.G.); 2 Division of Infectious Diseases and Immunology, University of Massachusetts Chan Medical School, Worcester, MA 01655, USA; jutamas.shaughnessy@umassmed.edu (J.S.); sanjay.ram@umassmed.edu (S.R.); 3 Department of Ophthalmology, University of Texas Southwestern Medical Center, Dallas, TX 75390, USA; 4 Department of Ophthalmology, University of Central Florida School of Medicine, Orlando, FL 32827, USA; 5 Department of Ophthalmology, Orlando College of Osteopathic Medicine, Orlando, FL 34787, USA

**Keywords:** glycobiology, alternative pathway, macrophages, microglia, treatment, ophthalmology, disease, geographic atrophy, immune system, inflammation

## Abstract

The alternative pathway of the complement system is implicated in the etiology of age-related macular degeneration (AMD). Complement depletion with pegcetacoplan and avacincaptad pegol are FDA-approved treatments for geographic atrophy in AMD that, while effective, have clinically observed risks of choroidal neovascular (CNV) conversion, optic neuritis, and retinal vasculitis, leaving room for other equally efficacious but safer therapeutics, including Poly Sialic acid (PSA) nanoparticle (PolySia-NP)-actuated complement factor H (CFH) alternative pathway inhibition. Our previous paper demonstrated that PolySia-NP inhibits pro-inflammatory polarization and cytokine release. Here, we extend these findings by investigating the therapeutic potential of PolySia-NP to attenuate the alternative complement pathway. First, we show that PolySia-NP binds CFH and enhances affinity to C3b. Next, we demonstrate that PolySia-NP treatment of human serum suppresses alternative pathway hemolytic activity and C3b deposition. Further, we show that treating human macrophages with PolySia-NP is non-toxic and reduces markers of complement activity. Finally, we describe PolySia-NP-treatment-induced decreases in neovascularization and inflammatory response in a laser-induced CNV mouse model of neovascular AMD. In conclusion, PolySia-NP suppresses alternative pathway complement activity in human serum, human macrophage, and mouse CNV without increasing neovascularization.

## 1. Introduction

**Figure 1:**
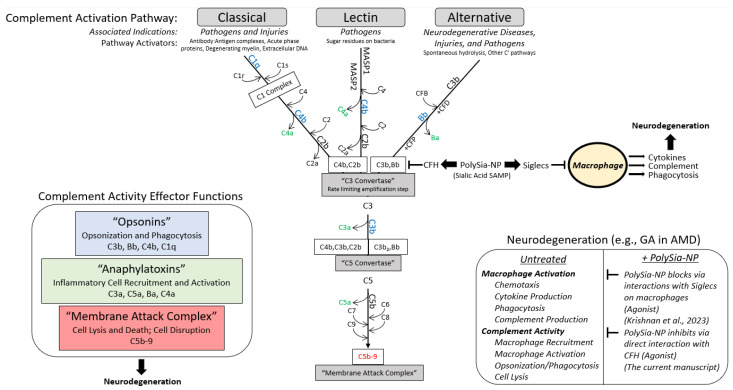
**Summary diagram.** Working hypothesis for PolySialic Acid-Nanoparticles (PolySia-NP)-mediated complement suppression mechanism of action. The complement system is a vital component of the innate immune system that can be activated [**top**] via 3 early pathways (classical, alternative, and lectin), which share a rate limiting step at the formation of C3 convertase [**middle**] and affect [**left**] opsonization and phagocytosis (opsonins in blue), macrophage recruitment and activation (anaphylatoxins in green), and cell lysis (membrane attack complex in red). The alternative pathway, which is associated with neurodegeneration [**right**], is protected from off-target, run-away activity by complement factor H (CFH) binding C3b to inhibit C3 convertase formation (reduces C3b binding to Bb) [**middle**]. In turn, CFH is regulated by binding of self-associated molecular patterns (SAMPs), including sialic acids, which enhance CFH binding to C3b. Therefore, PolySia-NPs were developed by Aviceda Therapeutics as improved sialic acid SAMPs to limit the propagation, amplification, and effects of the complement cascade in neurodegeneration [**middle** and **right**]. In addition, these PolySia-NPs have recently been reported to suppress macrophage activation, further protecting against inflammatory insults in neurodegeneration [**right**] [33].

The complement system is a zymogenic cascade of over 40 proteins that exert a potent host immune response to pathogens and are also present and active in tissue disease and injury (Figure 1) [1]. The effector functions of the classical complement cascade are largely achieved by stimulating inflammatory cells, particularly monocytes/macrophages/microglia, to migrate, proliferate, and phagocytose. In addition to their traditional role in host defense from pathogen infection, complement and myeloid cells exhibit diverse non-traditional functions in various diseases and injuries. In particular, neuro–immune interactions have critical roles in ophthalmologic and neurological disease progression. The complement system is pathogenic in a variety of body systems and indications, including acute sepsis [2,3], hematological diseases (paroxysmal nocturnal hemoglobinuria (PNH)) [2,3], auto-immune diseases (systemic lupus erythematosus and generalized myasthenia gravis (gMG)) [2], renal diseases (atypical hemolytic uremic syndrome (aHUS), C3 glomerulopathy, and IgA nephropathy) [2,3,4], organ transplantation rejection [2,3], trauma [2,3,5], ischemia/reperfusion injury [2,3,6], ischemic stroke [6], ophthalmic diseases (AMD, neuromyelitis optica spectrum disorder, and glaucoma) [2,3,7,8], neurodegenerative diseases (traumatic brain injury, spinal cord injury, Alzheimer’s disease, ALS, Huntington’s disease, and Parkinson’s disease) [2,3,5], fibrotic diseases [4], and periodontal diseases [9]. Consequently, complement has emerged as a major therapeutic target, and therapeutic modulation of complement has proven to be clinically successful for a variety of indications. For example, humanized monoclonal C5a antibodies eculizumab (Soliris, Alexion) and ravulizumab (Ultomiris, Alexion), which prevent C5 cleavage (and, therefore, C5a and C5b-9 production), are used for PNH, aHUS, gMG, and NMOSD [2].

Macular degeneration represents a leading cause of central vision loss in the elderly and is estimated to affect 288 million people worldwide [10]. Genomic and proteogenomic polymorphisms involved in regulation and modulation of the alternative complement pathway are closely associated with the development and progression of AMD [11,12,13,14]. The polymorphism associated with 50% of all cases of AMD is the Y402H polymorphism (rs1061170), which changes a tyrosine in the 402 amino acid position of CFH to a histidine [13]. This amino acid position corresponds to complement control protein domain 7 (CCP 7), a sialic acid/glycosaminoglycan binding region of CFH [14]. CCP domain 7 discriminates self from non-self by binding to polyanion patterns such as end-linked sialic acid glycan on the surface of host cells [15]. The Y402H polymorphism reduces the affinity of CCP domain 7 to these polyanions, reducing the ability of CFH to protect host cells from alternative complement attack [16]. This weakened ability to bind sialic acid self-markers contributes to chronic amplification of the alternative complement pathway and resultant chronic innate immune activation. With age, chronic inflammation leads to para inflammatory microglial overactivation, with eventual recruitment of peripheral-blood-monocyte-driven overt inflammation and development of late-stage complications of AMD such as geographic atrophy and exudative AMD [17].

Recently, intraocular complement factor depletion of C3 and C5 with intravitreally administered pegcetacoplan and avacincaptad pegol, respectively, demonstrated modest reductions in fundus-autofluorescence-defined geographic atrophy growth in dry AMD with a modest reduction in the rate of vision loss [18,19,20,21,22]. These modest benefits are accompanied by the risk of developing exudative AMD, optic neuritis, or blinding retinal vasculitis [19,20,22]. While the mechanism of these serious side effects continues to be investigated, there is pre-clinical evidence in a laser CNV model that profound knockout of C3 or C5 is pro-angiogenic and can stimulate the development of exudative AMD [23]. The role of complement in the maintenance and pruning of neurons would also be severely impaired by profound C3 inhibition, potentially leading to ischemic optic neuropathy reported in three phase-III clinical trial patients who received pegcetacoplan [19].

There are several pathways for complement activation (Figure 1). The classical complement pathway (CP) is activated when complement protein C1q binds to IgM or IgG antibody:antigen complexes, the surface of a pathogen, or acute phase proteins [1,24,25,26,27]. The lectin complement pathway is activated via binding of soluble defense lectins, which include mannose-binding lectin (MBL) and the ficolins, to sugar residues in a particular spacing common to bacterial pathogens. Alternative pathway (AP) complement activation occurs spontaneously by C3 hydrolysis, which constitutively produces C3b in a process called (“tick-over”) [28]. Unlike the self-limited activation of the classical and lectin pathway, alternative pathway activation is limited by CFH. Because of the potential for off-target cellular lysis, the formation of AP C3 convertase is tightly regulated by CFH. CFH is, in turn, regulated by its ability to bind sialic acid self-associated molecular patterns (SAMP), ligands for immune self-recognition receptors like CFH (the first described sialic acid SAMP receptor), which allow CFH to bind C3b and reduce its ability to bind Bb [29,30,31,32]. When CFH is not bound to a SAMP, C3b binds Bb with high affinity and results in the propagation and amplification of membrane attack complex formation and cellular lysis of host and pathogen cells alike. Sialic acids, including PSA, can suppress complement-associated inflammation both by binding Siglecs on inflammatory cells and by binding CFH. Recently, we have demonstrated that a nanoparticle coated with Poly Sialic acid, “PolySia-NP”, was able to mimic sialic acid SAMP, which agonized Siglecs (sialic-acid-binding, immunoglobulin-like lectins), self-associated pattern recognition receptors, to resolve macrophage polarization [33]. These PolySia-NPs could also be used to present sialic acid ligands to mimic SAMPs. In this paper, we extend these findings to PolySia-NP-mediated suppression of complement activity. In contrast to C3 or C5 depletion, this Sialic acid–CFH-mediated attenuation of the alternative complement pathway results from competitive binding of C3b or C3 convertase (C3bBb) with factor B [34]. The enhanced binding of both C3b and C3bBb by Sialic acid–CFH complex inhibits binding of or displaces Bb from C3b, preventing the amplified formation of C3a, C5a, and C5b-9 and cellular lysis and inflammatory activation [35].

The inhibition of alternative pathway complement amplification through PolySia-NP-CFH should permit classical, lectin, and constitutive AP pathway activation. In contrast, complement factor depletion strategies will inhibit all three complement pathways along with constitutive alternative pathway activation. CFH-mediated AP pathway attenuation should not immunocompromise, should permit complement maintenance functions, and should not induce neovascularization. As a direct C3 inhibitor, PolySia-NP should be equivalent to pegcetacoplan in attenuating the AP pathway without the potential side effects resulting from depleting complement factors that disable all three complement pathways.

In this paper, we demonstrate that PolySia-NPs developed by Aviceda Therapeutics (Cambridge, MA, USA) can dampen the alternative complement cascade directly by binding and activating CFH, increasing CFH binding affinity to C3b, and attenuating C3 cleavage, leading to physiologic suppression of the alternative complement pathway in vitro and in vivo. In this manuscript, we use several in vitro models and an in vivo disease model to assay complement binding and activity. This work is the first to demonstrate complement regulation using a sialic-acid-containing nanoparticle formulation. Combined with our recent work with this PolySia-NP demonstrating resolution of disease-associated pro-inflammatory macrophages [33], these new findings suggest a potential multi-pronged anti-inflammatory therapy relevant to AMD and the many other neurodegenerative diseases for which both cellular and humoral inflammatory overactivation are involved. This work is valuable to the scientific and medical community for both its therapeutic potential and the novel insights into sialic acids in complement regulation.

## 2. Results

### 2.1. PolySia-NPs Interact with Alternative Complement Inhibitor CFH

**Figure 2:**
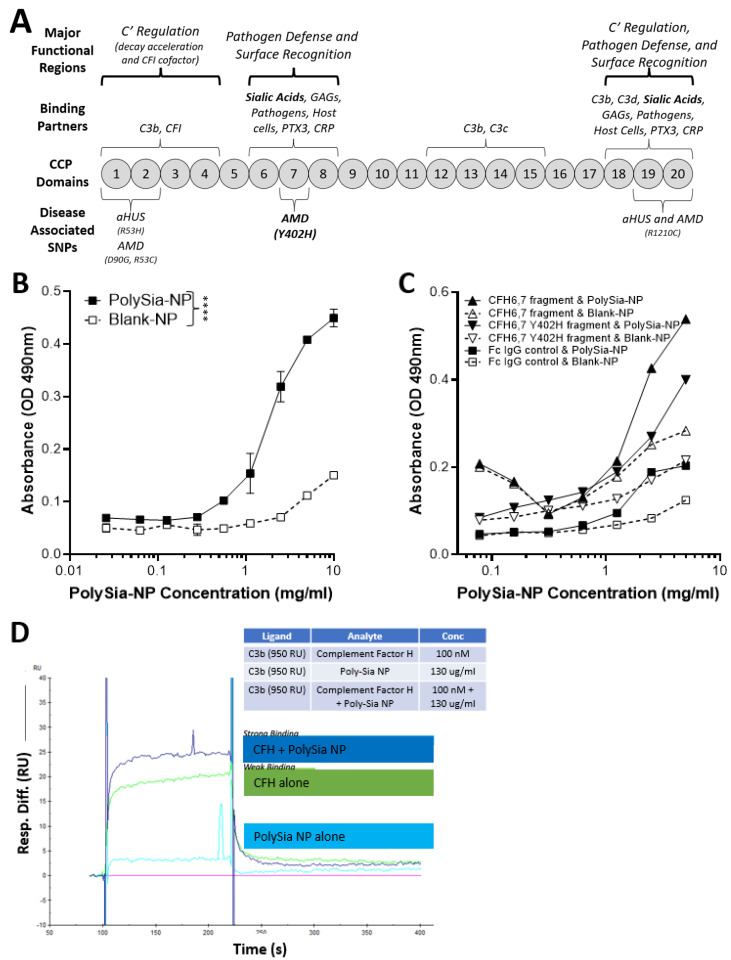
**Complement factor H (CFH) and PolySia-NPs.** PolySialic acid nanoparticles bind to complement inhibitor CFH and enhance binding of CFH to C3b. (**A**) Simple diagram of CFH structure with selected binding domains and disease-associated SNPs. CCP domains 6–7 and 19–20 bind sialic acid to regulate complement activation, and CCP domain 7 contains the most common AMD-associated polymorphism (Y402H). (**B**) PolySia-NP bound directly to CFH (C; IC50 = 2 mg/mL) in an assay in which CFH-His or fragment CFH-IgG was bound to appropriate plates, incubated with PolySia-NP or control NP, labeled with biotinylated PEG antibody, and absorbance quantified (N = 4 independent experiments; **** *p* < 0.0001 2 way ANOVA treatment main effect). (**C**) PolySia-NP also bound directly to CFH fragments of CCP domains 6–7, both wild-type and Y402H polymorphism. (**D**) Biacore binding assay demonstrated increased binding of CFH to C3b in the presence of PolySia-NP (“CFH + PolySia-NP”; dark blue line), versus without PolySia-NP (“CFH alone”; green line) and PolySia-NP alone (light blue line).

CFH is a complement regulatory protein consisting of 20 short consensus repeat domains of approximately 60 amino acids in length called complement control protein (CCP) domains (Figure 2A). CCP domains 1–4 and 19–20 bind C3b (alternative pathway inhibition), and CCP domains 18–20 and CCP domain 7, sialic acid, GAG, and heparin binding regions. Sialic acid binding to domain 7 serves as a “proof reader” to discriminate between self and non-self patterns. CCP 7 is also the location of the common polymorphism (35% of people with European descent) strongly associated with increased risk for AMD (2.3×–5.2×) [15]. Binding of both sialic acid domains is required to increase the affinity of CFH to C3b [15].

To determine if PolySia-NP binds to CFH directly (Figure 2B), we first coated plates with human CFH-His, then incubated PolySia-NP at various concentrations (0.3–10 mg/mL) and finally detected the PolySia-NP using a biotinylated antibody against the PolySia-NP’s polyethylene glycol (PEG) linker. The resulting binding data demonstrated an S-shaped binding curve with an IC50 of approximately 2 mg/mL, therefore confirming dose-dependent binding of PolySia-NP to human CFH in vitro. To determine the location of PolySia-NP binding on CFH, we similarly tested CFH wild-type and Y402H polymorphism fragments of CCP domains 6–7 and found evidence of PolySia-NP binding to both wild-type and AMD-associated polymorphism CFH fragments from this region (Figure 2C).

To test whether PolySia-NP binding to CFH could enhance CFH-mediated complement inhibition by increasing CFH binding to C3b, a Biacore assay was performed (Figure 2D). C3b was immobilized to Fc2 at 1600 RU and Fc3 at 1450 RU and 950 RU, and the following binding conditions were tested: buffer alone, human CFH, and human CFH + PolySia-NP. When PolySia-NP was incubated with CFH, increased binding of Polysia-NP-CFH to C3b was observed compared to CFH without Polysia-NP. These data demonstrate that PolySia-NP interacts with CFH to increase affinity binding to C3b. The increased binding of PolySia-NP-CFH to C3b should displace Bb in the formed c3 convertase (C3bBb) or prevent Bb from binding C3b to form C3 convertase. These results suggest that PolySia-NP does interact with CFH to down-regulate the alternative pathway of complement by preventing the formation or degradation of C3 convertase.

### 2.2. PolySia-NPs Suppress Complement Activity in Human Serum: Hemolysis

**Figure 3:**
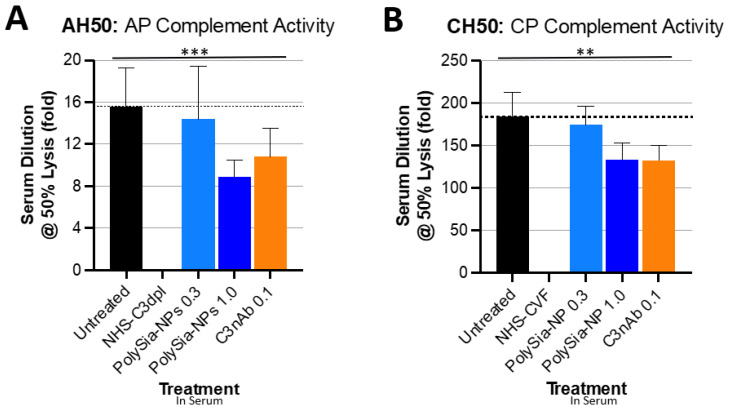
**PolySia-NPs affect complement activity in hemolytic assays.** PolySialic acid nanoparticles decrease alternative and classical complement hemolytic activity in response to human serum. (**A**) PolySia-NP treatment of human serum moderately protects from alternative pathway hemolytic response (similar effect size as C3b neutralizing antibody) as measured using AH50 assay (N = 3 independent experiments; *** *p* < 0.001 2 way ANOVA treatment main effect). (**B**) PolySia-NP treatment of human serum modestly protects from classical pathway hemolytic response (similar effect size as C3b neutralizing antibody) as measured using CH50 assay (N = 6 independent experiments; ** *p* < 0.01 2 way ANOVA treatment main effect). NHS-C3dpl = normal human serum with C3 depletion; NHS-CVF = normal human serum pretreated with cobra venom factor; C3nAb = C3 neutralizing antibody.

To demonstrate the ability of PolySia-NP to actuate CFH inhibitory regulation of the alternative complement pathway, we tested the potential for PolySia-NPs to suppress alternative pathway complement activity in human serum using an AH50 assay (Figure 3A), in which normal human serum was spiked with either nothing (untreated normal activity control), PolySia-NP (0.3 mg/mL, 1 mg/mL), or commercially available C3 neutralizing antibody (C3nAb), then immediately assayed for hemolytic activity of rabbit erythrocytes. Human serum with C3 depleted (NHS-C3dpl) was included as a very low activity control. As shown in Figure 3A, PolySia-NPs appear to modestly decrease serum hemolytic AP activity in a dose-dependent manner, as predicted, with a similar effect size as C3nAb.

We then assessed the potential for PolySia-NP to affect the classical pathway (CP) of complement activity. Although direct PolySia-NP interaction with CFH is not predicted to affect the classical pathway, per se, indirect effects of PolySia-NP on classical cascade amplification are possible. We, therefore, tested the potential for PolySia-NPs to suppress classical pathway complement activity in human serum using the CH50 assay (Figure 3B), in which normal human serum was spiked with IgM (potent activator of CP) and either nothing (untreated normal activity control), PolySia-NP (0.3 mg/mL, 1 mg/mL), or commercially available C3 neutralizing antibody (C3nAb), then immediately assayed for hemolytic activity of antibody-sensitized sheep erythrocytes. Human serum pretreated with cobra venom factor (NHS-CVF) to consume complement was included as a very low activity control. As shown in Figure 3B, PolySia-NPs appear to modestly decrease serum hemolytic CP activity in a dose-dependent manner, as predicted, with a similar effect size as C3nAb.

Although not directly relevant to the pathology of AMD and neurodegenerative diseases (which do not involve pathological lysis of red blood cells), hemolytic assays are the long-term gold standard and most common test for complement activity in both clinical and research labs. These assays, the CH50 for classical pathway and the AH50 for alternative pathway, test competency of complement-containing human serum for hemolysis of red blood cells by C5b-9, both normally and following treatment predicted to affect complement activity. Hemolytic assays have been used to demonstrate CFH-mediated complement regulation and the impact of SNPs in CFH, associated with disease [36,37]. We used these assays because of their ubiquity and as an initial assessment of the effect of PolySia-NP on general complement activity by pathway, rather than to study complement-mediated hemolysis, per se.

### 2.3. PolySia-NPs Suppress Complement Activity in Human Serum: Opsonization (C3b Deposition)

The presence of C3 fragments (cleavage products) indicates complement activity and is relevant to AMD and neurodegenerative disease pathology, where anaphylatoxin fragments (C3a) recruit and activate inflammatory cells and opsonin fragments (C3b, iC3b, C3d, and C3c) mark local tissues for inflammatory attack, resulting in local damage. Complement deposition assays in human serum are frequently used to assess this disease-associated complement opsonization activity, including in a pivotal study that reported complement inhibition in this assay using PolySia alone [36,38].

**Figure 4:**
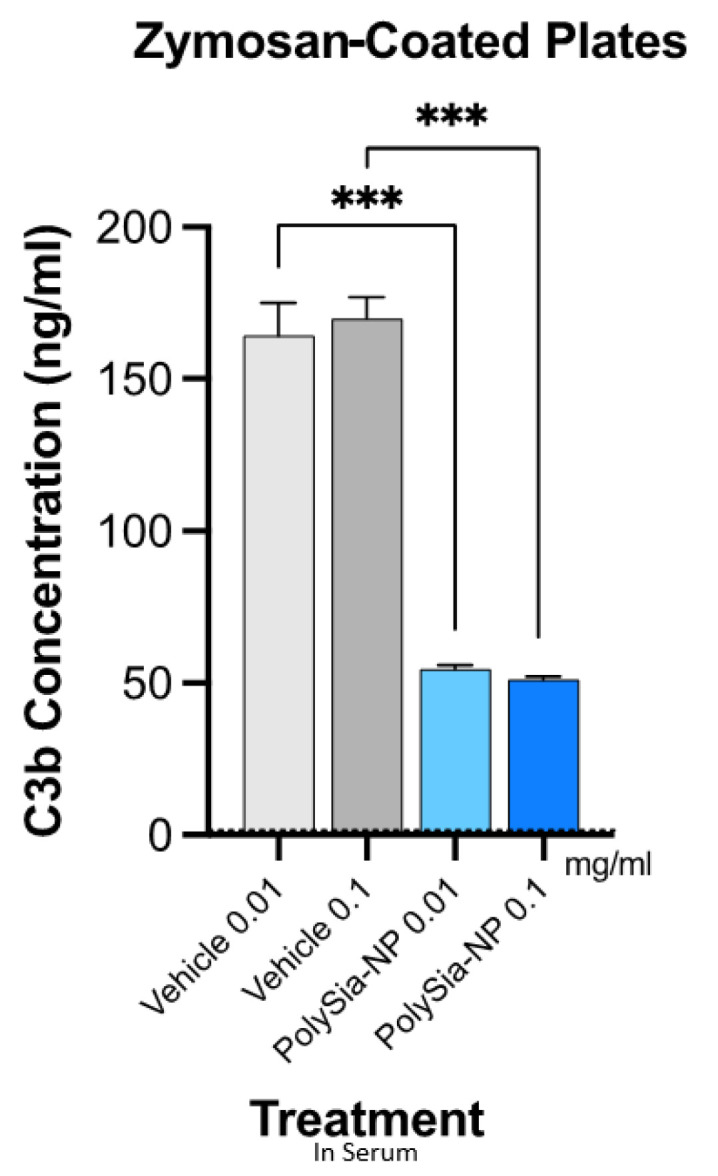
**PolySia-NPs inhibit alternative-pathway-mediated deposition of C3b in human serum.** PolySia-NP incubation on plates coated with Zymosan (activator of alternative pathway) strongly reduced complement opsonin C3b deposition from serum. *** *p* < 0.001 *t*-test PolySia-NPs vs. vehicle controls.

We, therefore, next tested the potential for PolySia-NPs to suppress alternative pathway complement activity in human serum using the serum C3b deposition assay (Figure 4), in which normal human serum was spiked with Zymosan (yeast cell wall preparation; potent AP activator) and either vehicle (10% sucrose control) or PolySia-NP (0.01 mg/mL, 1 mg/mL), and C3b deposition onto the well surface was quantified. As shown in Figure 4, PolySia-NPs substantially and significantly decrease C3b deposition from human serum. PolySia-NP-mediated complement inhibition appears to be equal to or stronger than that of PolySia alone [38].

Taken together, these data suggest that PolySia-NP attenuates complement pathways, especially the AP, in serum-based assays.

### 2.4. PolySia-NPs Suppress Complement Activity in Human Macrophages In Vitro

**Figure 5:**
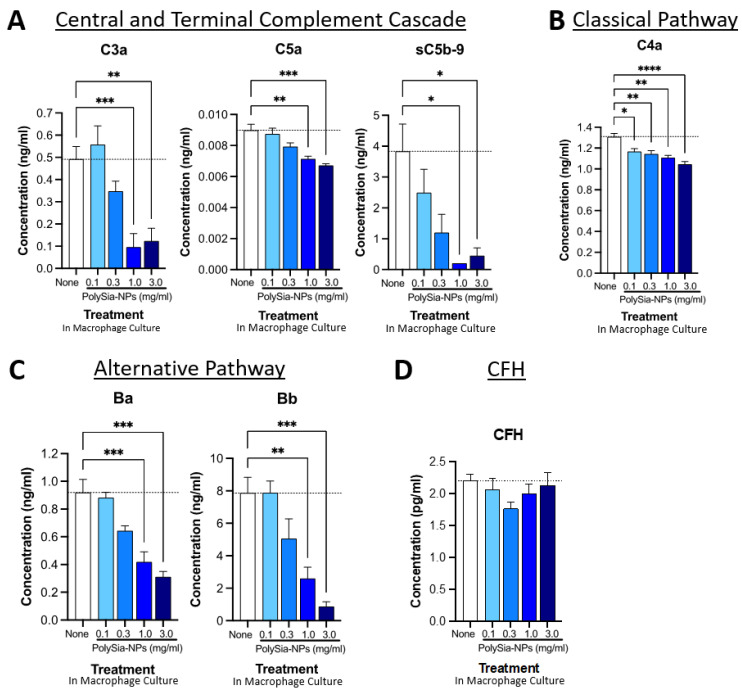
**PolySia-NPs affect complement activity in human macrophages.** PolySia-NP treatment of cultured human PBMC-derived M1 macrophages decreased complement activation markers (e.g., anaphylatoxins), mainly through inhibition of the alternative pathway, as measured using complement multiplex ELISA. (**A**) Treatment of macrophages with PolySia-NP decreased C3a and C5a anaphylatoxin and sC5b-9 soluble MAC production of the central and terminal complement cascades, respectively. Treatment of macrophages with PolySia-NP slightly decreased classical pathway C4a anaphylatoxin levels (**B**) and strongly decreased levels of alternative pathway activation fragments Ba and Bb (**C**). (**D**) Treatment of macrophages with PolySia-NP did not substantially affect alternative pathway inhibitor CFH levels. * *p* < 0.05, ** *p* < 0.01, *** *p* < 0.001, **** *p* < 0.0001 1way ANOVA Dunnett’s post-test PolySia-NP vs. untreated control (“None”).

We next used cell-based experiments to investigate the complement inhibitory function predicted from the cell-free experiments (Figure 5). In cell-based experiments, we predict that PolySia-NPs will directly interact with CFH to decrease complement activation. In addition, we predict that PolySia-NPs may indirectly decrease complement activation through PolySia-NP’s interaction with macrophages. As described in our recent paper [33], PolySia-NPs reduce pro-inflammatory polarization (promote resolution), and so would also be expected to reduce complement protein production and activation by macrophages. Monocytes and stimulated (e.g., polarized) macrophages are capable of producing most complement proteins, including functional classical, alternative, and terminal pathways [39,40]. As explained above, all complement pathways share common downstream effectors, including C3, C5, and C5b-9, and complement activation can be detected by increased anaphylatoxin (e.g., C3a and C5a) and soluble membrane attack complex (sC5b-9) levels.

We began by testing the effect of PolySia-NP on central and terminal pathway complement effectors C3a, C5a, and sC5b-9 levels in human PBMC-derived M1 macrophage cultures using multiplex ELISA (Figure 5A). Following 24 h treatment with various concentrations of PolySia-NPs (0.1–3.0 mg/mL) or control, we detected dose-dependent decreases in all three activation analytes in the macrophage supernatant. These results support the ability of PolySia-NP to therapeutically suppress central and terminal pathway complement activity in macrophages.

We then conducted a similar analysis of the classical pathway effector C4a (Figure 5B), which indicated a modest suppression of the classical pathway. Next, we conducted a similar analysis of the alternative pathway effectors Ba and Bb (Figure 5C), which indicated a moderate suppression of the classical pathway. Finally, we verified that the amount of CFH was not affected by PolySia-NP treatment (Figure 5D), only its binding and associated function.

We also extended these findings to the THP-1 human macrophage cell line (Supplementary Figure S1). In THP-1 cells, PolySia-NP treatment similarly does not appear to affect CFH levels but modestly decreases the levels of both complement factor D (CFD; cleaves CFB to effectors Ba and Bb) and complement factor P (properdin; C3-convertase-stabilizing protein). Taken together, these data suggest that PolySia-NP reduces complement activity, especially the AP, in cell-based assays.

### 2.5. PolySia-NP Treatment Is Not Cytotoxic to Cultured Macrophages, Microglia, and Neural Cells

**Figure 6:**
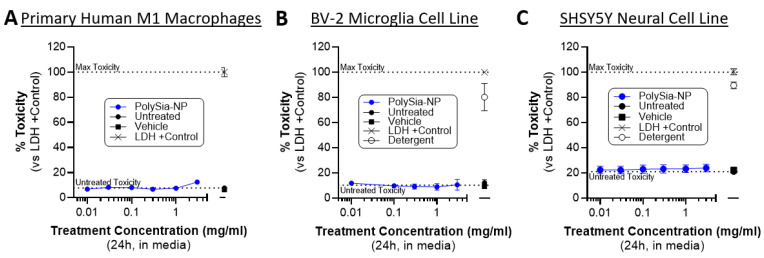
**PolySia-NPs are non-toxic to macrophages, microglia, and neural cells.** CytoTox96 assays on supernatants from PolySia-NP treated cell cultures (1 day treatment) confirm that PolySia-NP is not directly toxic to macrophages (N = 1 experiment) (**A**), microglia (N = 4 independent experiments) (**B**), or neural cells (N = 3 independent experiments) (**C**), at any dose tested (0.01–3.0 mg/mL). Dotted lines show controls: top dotted line represents max toxicity and bottom dotted line represents untreated toxicity.

Next, we confirmed that PolySia-NP treatment is non-cytotoxic to relevant cells in vitro. Our working hypothesis is that PolySia-NP mediates complement inhibition directly through CFH and likely also indirectly by signaling though Siglecs on macrophages, leading to suppression of pro-inflammatory signaling and promotion of inflammatory resolution, as we previously described for macrophage inflammation and cytokine production [33]. An alternative hypothesis for some of the findings in the cell-based experiments above (Figure 6) is that PolySia-NP is cytotoxic to the tested inflammatory cells. Therefore, we performed CytoTox96 lactate dehydrogenase (LDH)-based toxicity assays on supernatants from cultured human M1 macrophages treated with PolySia-NP (0.01–3.0 mg/mL) or negative controls (untreated, vehicle) for 1 day or positive controls (LDH, detergent) for 30 min. As shown in Figure 6A, all tested concentrations of PolySia-NP are non-toxic to these cells. These data agree with and extend our previous study, which showed no decrease in the viability of human macrophages treated with PolySia-NP [33]. We next extended these non-cytotoxic findings to microglia, a local complement-producing and complement-responding inflammatory cell type that is also implicated in AMD and many other ophthalmological and neurological diseases using the CytoTox96 assay on the mouse BV-2 microglia cell line (Figure 6B).

For therapeutic application to ophthalmological and neurological tissues, it is important to limit toxicity to the resident non-inflammatory cells. Therefore, we used the CytoTox96 assay to test for PolySia-NP toxicity on the human neural cell line SHSY5Y (Figure 6C). In line with our other published studies and in preparation work on PolySia-NP safety in multiple in vitro and in vivo models including mouse eye and rabbit eye [33], we do not observe any toxicity in this assay (Figure 6C).

### 2.6. PolySia-NP Treatment Reduces Microglia and Complement Response to Laser CNV In Vivo

**Figure 7:**
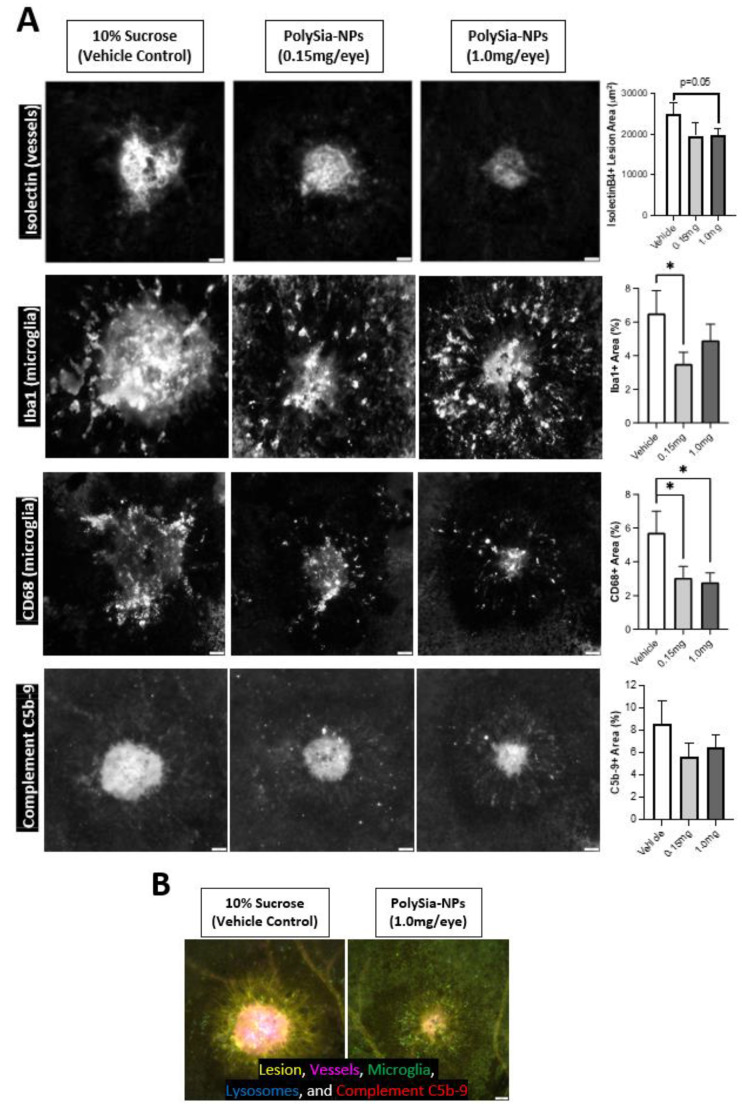
**Microglia and complement in mouse CNV model.** Intravitreal PolySia-NP treatment attenuates microglia and complement inflammatory responses 8 days after laser-induced choroidal neovascularization (CNV) in vivo. (**A**) Representative images and graphs of choroid laser injury sites fluorescently immunolabeled for isolectinB4 (top row; lesion and blood vessel marker), Iba1 (2nd row; microglia marker), CD68 (3rd row; phagocytosis and microglial activation marker), and C5b-9 (bottom row; terminal complement complex). N = 16–20 lesions (5 mice) per group. These data demonstrate that PolySia-NP treatment inhibited both neovascularization and inflammatory response in this model. * *p* < 0.05 *t*-test PolySia-NP vs. vehicle control. (**B**) Single representative multichannel image of choroidal injury site in vehicle-treated versus PolySia-NP treated mice.

To confirm these findings in vivo, we utilized a humanized Siglec-11 transgenic mouse model in a laser-induced choroidal neovascularization (laser CNV) model of wet AMD (Figure 7). In this disease model, laser-injury of the choroid induces inflammation, neovascularization, and visual deficits. Mice were treated with PolySia-NP (low or high dose) or vehicle control (10% sucrose) at the time of laser CNV induction and euthanized 8 days later; immunohistochemical analysis of blood vessels (Isolectin-B4), pro-inflammatory monocytes (Iba-1 and CD68), and complement (C5b-9) markers was performed on the laser injury sites in choroidal tissue.

Importantly, mouse Siglec biology is different and less complex than that in humans. One difference is that mice lack an ortholog to human Siglec-11, which binds preferentially to α2–8-linked sialic acids and is expressed in tissue macrophages and microglia; our transgenic mouse model expresses humanized Siglec-11 in addition to murine Siglec orthologs. This Siglec-11 humanized mouse model enables valuable in vivo experiments, but because it does not fully recapitulate human Siglec biology, we expect PolySia-NP to exhibit modest effects in mice relative to human cells in vitro and patients in the clinic.

As shown in Figure 7, PolySia-NPs attenuate neovascularization after laser CNV in Siglec-11 transgenic mice as assessed via isolectin immunohistochemistry. For detailed data and analyses of neovascularization and function in this study, and for a similar study featuring non-mutant mice and a blank-NP control, see Krishnan et al., 2023 [33]. Importantly, as predicted by the data in Krishnan et al., 2023, PolySia-NPs also attenuate the microglial response to laser CNV, and their phagocytic activation, as visualized and assessed using Iba-1 and CD68 immunohistochemistry, respectively. Notably, this injury-induced increase in monocyte activation would be predicted to increase local complement protein release and activation. Finally, as predicted based on the data presented in Figure 2, Figure 3, Figure 4, Figure 5 and Figure 6 of this paper, PolySia-NP treatment trends to modestly decrease C5b-9 deposition in laser CNV sites. Together, these data highlight the potential therapeutic potential for PolySia-NP to treat diseases and injuries associated with damaging pro-inflammatory monocytes and complement activity.

## 3. Discussion

The binding of PolySia-NP to CFH, demonstrated using three separate assays, confirms the hypothesis by Meri and Pangburn that a three-dimensional requirement is necessary for sialic acid structures to bind and actuate CFH [35]. This hypothesis explained why linear polysialic acid or coliminic acid alone was not able to bind or actuate CFH [14,41]. Sialic acid self-associated molecular patterns (SAMPs) were first described by Ajit Varki who used the example of CFH as the first sialic acid SAMP recognition receptor [32]. Sialic acid SAMP recognition receptors, by definition, recognize a pattern of end-linked sialylated glycans, not just a single glycan or a linear polymer. Presenting a pattern of PSA glycans on the surface of a nanoparticle, binding and actuation of CFH was enabled.

In vivo, CFH must bind a SAMP presented by host cells to conformationally change CFH and enhance C3b binding. CFH competes with Bb to bind C3b, so higher affinity binding of PolySia-NP-CFH will displace Bb, thereby preventing or decaying the formation of C3bBb (C3 convertase) with resultant decay dissociation of amplification C3 convertase of the alternative pathway [34]. The data presented here demonstrate that PolySia-NP can behave as a SAMP and attenuate the alternative complement cascade by directly regulating CFH.

Recently FDA-approved complement pathway inhibitory treatment options for non-exudative AMD utilize complement factor 3 and 5 depletion, which validates complement inhibition as a viable therapeutic strategy [42]. While there are no validated clinically translated models for non-exudative macular degeneration, the utilization of the laser induced neovascular model has been shown to be CFH dependent, implicating the alternative complement pathway in this model’s etiogenesis [43]. Furthermore, other laboratories have shown the ability of PSA alone to be able to attenuate this model with the suppression of the complement pathway via degradation of C3 convertase and attenuation of activated microglia [38]. In our study, we demonstrated the ability of PSA-NP to attenuate the alternative complement pathway in vivo in a clinically relevant model of AMD utilizing a non-complement-factor-depleting method.

Depletion of C3 or C5 completely disables all three complement pathways, which carries significant theoretical risks. On the other hand, attenuation of the AMD-associated alternative complement pathway without disabling the whole complement system could provide a safer inhibition strategy. For example, recombinant CFH injection therapy, which represents this more focused strategy, was demonstrated to be safe in a clinical trial [44]. The complement system is the first line of defense against bacterial infections and profoundly disabling classical and lectin pathways could lead to an increased risk of endophthalmitis—a complication seen in clinical trials of pegcetacoplan [45,46]. The alternative complement cascade in coordination with microglia carry out neuronal pruning and maintenance; if the AP is completely disabled, it could result in damage to the optic nerve. In a C3 knockout mice strain, amyloid beta accumulation on the Bruch’s membrane was reported, along with increased levels of retinal TNF-alpha and calcitonin, pointing to an important role of C3 in maintaining retinal homeostasis [47]. Spontaneous inflammation seen in C3 knockout mice may play a role in the pathogenesis of retinal vasculitis seen clinically in patients receiving intravitreal injections of a profound C3 depleter pegcetacoplan [20].

The conversion to the exudative form of AMD may also represent a complication of complement factor depletion as evidenced by pre-clinical studies in the laser-induced CNV model of wet macular degeneration. Laser-induced choroidal neovascularization was enhanced in both C3 and C5 knockout mice, implicating C3 and C5 as having a potential anti-angiogenic function [23]. To determine if PolySia-NP-regulated CFH alternative pathway attenuation would result in increased angiogenesis, we tested PolySia-NP in a laser-induced CNV model and demonstrated not only an abrogation of the formation of the membrane attack complex but also a reduction in neovascularization.

To our knowledge, this is the first demonstration of alternative complement attenuation through the actuation of CFH decay accelerating activity. By mimicking sialic acid self-associated molecular patterns, which are the endogenous checkpoint agonists for complement, PolySia-NP is able to physiologically resolve complement amplification. This actuation requirement by CFH can explain why delivery of recombinant CFH failed to advance clinically and demonstrated no clinical efficacy in early clinical trials. CFH alone without actuation by the correct sialic acid SAMP will be unable to decay C3 convertase and de-amplify the AP.

## 4. Materials and Methods

### 4.1. Animals

The in vivo laser CNV study used 15 adult (male and female) humanized Siglec-11 transgenic mice (N = 5/group), as previously described [33]. The humanized Siglec-11 transgenic mice were generated at Cyagen (Santa Clara, CA, USA) via human SIGLEC-11 (NCBI Reference Sequence: NM_052884.3) knock-in at the locus of ROSA26 gene (NCBI Reference Sequence: NR_027008.1) in C57BL/6N mice by CRISPR/Cas-mediated genome engineering. For the knock-in, the “CAG promoter-Kozak-human SIGLEC11 CDS-rBG pA” cassette was cloned into intron 1 of ROSA26 in reverse orientation. To engineer the targeting vector, homology arms were generated by PCR using BAC clone as template. Cas9 and gRNA were co-injected into fertilized eggs with targeting vector for the production of mice. Mice were bred and housed at Taconic Biosciences (Rensselaer, NY, USA) and then shipped to Powered Research CRO (Durham, NC, USA) for the experiment.

### 4.2. PolySia-NP

Poly Sialic Acid (PolySia) Nanoparticles (NPs), “PolySia-NPs”, were prepared by Aviceda Therapeutics, as previously described [33,48]. In brief, PolySia-NPs were prepared using a core of polyethylene glycol (PEG) and poly-lactic co-glycolic acid (PLGA) copolymers to which PolySia was covalently attached.

Conjugation step: PolySia-NPs were manufactured by first covalently conjugating PLGA-PEG polymer and PSA (polysialic acid). A polymer phase was prepared by dissolving PLGA-PEG polymer in a suitable vehicle. Separately, the PSA phase was prepared, where PSA was dissolved in suitable vehicle. Both phases were mixed and kept for conjugation for a pre-defined time. This is identified as the organic phase during manufacturing. Separately, an aqueous phase was prepared.

Nanoparticle formation: A primary coarse suspension was formed by mixing the organic phase to aqueous phase. The suspension was further processed via high pressure homogenizer to reduce the final average particle size to ~100 nm. The resulting particles were purified using a filtration process to remove excess solvents and undesired process impurities. The purified suspension was then subjected to tonicity and pH adjustment, pre-filtration, followed by filtration through a 0.2-micron filter under laminar flow.

### 4.3. Binding Assays

#### 4.3.1. Biacore

Biacore assay was performed to assess binding interactions between PolySia-NP, CFH, and C3b. C3b was immobilized to Fc2 at 1600 RU and Fc3 at 1450 RU and 950 RU. Groups were as follows: buffer alone, human complement factor H at 200 nM/100 nM, and human CFH + PolySia-NP at 1:20 dilution (10 min room temperature incubation).

#### 4.3.2. ELISA-like

CFH-His (1 μg/mL; Abcam, Cambridge, UK, or Cloud Clone, Katy, TX, USA) was bound to nickel-coated plates (Thermo Fisher Scientific, Waltham, MA, USA) via overnight incubation at RT. CFH wild-type and Y402H polymorphism fragments (FH6,7/hIgG1Fc and FH6,7 Y402H/hIgG1Fc; 1 μg/mL each), generated as previously described [49,50], were bound to ELISA plates (R&D Systems, Minneapolis, MN, USA) via overnight incubation at RT. Negative controls included PBS and recombinant human IgG-Fc (R&D Systems). Coated wells were washed and then PolySia-NPs (0.3–10 mg/mL) or blank NP negative control were incubated for 2 h, followed by washing, biotinylated PEG antibody (0.5 μg/mL; Abcam, Waltham, MA, USA) incubation for 1 h, washing, and streptavidin-HRP (R&D Systems) incubation for 20 min. PEG was then visualized using TMB substrate (R&D Systems) reaction, stopped (R&D Systems), and read at 490 nm absorbance using a BioTek Synergy HTX plate spectrophotometer. Four independent experiments were performed and data were combined for presentation here.

### 4.4. Complement Hemolytic Assays

#### 4.4.1. CH50

Normal human serum (NHS; Complement Technology, Tyler, TX, USA) was treated with either nothing (untreated), sucrose vehicle control, PolySia-NP (0.3 mg/mL, 1 mg/mL), or C3 neutralizing antibody (C3nAb; Millipore, Burlington, MA, USA) and immediately used for CH50 assays. Normal human serum with cobra venom factor pre-activation (NHS-CVF) was included as a negative control. In brief, antibody-sensitized sheep erythrocytes (Complement Technology) were added to a dilution series of each serum sample in GVB++, incubated at 37 °C for 60 min, centrifuged to pellet remaining RBCs, transferred supernatants (containing heme from lysed RBCs) to 96-well plates, and read on a BioTek Synergy HTX plate spectrophotometer (Agilent, Lexington, MA, USA) at 560 nm absorbance. All data were normalized to the positive control at 100% (replace buffer with water or detergent) and negative control at 0% (no NHS). Six independent experiments were performed, and data were combined for presentation here.

#### 4.4.2. AH50

Normal human serum (NHS, Complement Technology) was treated with either nothing (untreated), sucrose vehicle control, PolySia-NP (0.3 mg/mL, 1 mg/mL), or C3 neutralizing antibody (C3nAb, Millipore) and immediately used for AH50 assays. Normal human serum with C3 depletion (NHS-C3dpl, Complement Technology) was included as a negative control. In brief, rabbit erythrocytes (Complement Technology) were added to a dilution series of each serum sample in GVB plus MgEGTA, incubated at 37 °C for 30 min, centrifuged to pellet remaining RBCs, transferred supernatants (containing heme from lysed RBCs) to 96-well plates, and read on a BioTek Synergy HTX plate spectrophotometer at 560 nm absorbance. All data were normalized to positive control at 100% (replace buffer with water or detergent) and negative control at 0% (no NHS or add EDTA). Three independent experiments were performed, and data were combined for presentation here.

### 4.5. Complement Opsonization/Deposition Assay

Wells were blocked (1% bovine serum albumen (BSA)), followed by 3 h incubation with zymogen (potent activator of the alternative complement pathway) in defibrinated plasma (serum), plus PolySia-NP or vehicle (10% sucrose) control. Complement deposition was visualized after 1 h incubation with human C3b antibody (Complement Tech-A114), then 20 min streptavidin-HRP (R&D Systems) incubation, and 20 min TMB substrate (R&D Systems) reaction. Results were read using a BioTek Synergy HTX plate spectrophotometer (Agilent) at 490 nm absorbance.

### 4.6. Cells

#### 4.6.1. Primary M1 Human Macrophages

Primary human macrophages were derived from primary human blood mononuclear cells (PBMCs), which were obtained from fresh 1/4 pack Leukopak^®^ (Stemcell Technologies, Cambridge, MA, USA). Monocytes were isolated and enriched using CD14+ magnetic beads from EasySep Human Monocyte Isolation kit (Stemcell Technologies). Monocyte purity was confirmed using flow cytometry. The CD14+ enriched cells were frozen and stored in liquid nitrogen until use. Approximately 6 days prior to experimental treatment, the cryopreserved monocytes were thawed and cultured in 96-well plates at 250,000 cells/mL in serum-free ImmunoCultTM-SF Macrophage Differentiation Medium (Stemcell Technologies) with 50 ng/mL macrophage colony stimulating factor (M-CSF), which differentiated the monocytes into macrophages. Approximately 4 days after plating, macrophages were activated to the M1 state using LPS (10 ng/mL; Sigma Aldrich, St. Louis, MO, USA) and interferon-gamma (IFN-γ; 50 ng/mL; Stemcell Technologies).

#### 4.6.2. THP-1 Cell Line

The human leukemia monocytic cell line THP-1 (ATCC TIB-202TM, Gaithersburg, MD, USA) was cultured and expanded for experiments in 87% RPMI media (Thermo Fisher Scientific), plus 10% fetal bovine serum, 1% penicillin-streptomycin, 1% L-glutamine, and 1% sodium pyruvate, with enzymatic passaging using trypsin. THP-1 monocytes were plated onto 96-well culture plates at 50,000–100,000 cells/well and differentiated to macrophages for 2 days using 10 ng/mL phorbol 12-myristate 13-acetate (PMA) in RPMI with 1% penicillin-streptomycin, 1% L-glutamine, 1% sodium pyruvate, 1% N2 supplement, and 1% chicken serum. THP-1 monocytes were then activated using LPS (10 ng/mL; Sigma) for 1 day before treatments were applied.

#### 4.6.3. BV-2 Cell Line

The immortalized mouse microglial cell line BV-2 (CLS305156; Cell Lines Service, Sioux Falls, SD, USA) was cultured and expanded for experiments in RPMI media (Thermo Fisher Scientific), plus 10% fetal bovine serum and 1% penicillin-streptomycin, with enzymatic passaging using TrypLE Select (Thermo Fisher Scientific). BV-2 microglia were plated onto 96-well culture plates at 25,000–50,000 cells/well and grown for 1–2 days before activation with LPS (0.1–1.0 μg/mL; Sigma Aldrich) and treatment addition.

#### 4.6.4. SHSY5Y Cell Line

The human neuroblastoma-derived cell line SHSY5Y (ATCC CRL-2266; ATCC, Gaithersburg, MD, USA), a popular albeit imperfect neuronal model, was cultured and expanded for experiments in 42% Ham’s F12 media (Sigma Aldrich), plus 42% EMEM media (Sigma Aldrich), 15% fetal bovine serum, 1% penicillin-streptomycin, and 1% non-essential amino acids, with enzymatic passaging using TrypLE Select (Thermo Fisher Scientific). SHSY5Y neural cells were plated onto 96-well culture plates at 25,000 cells/well and grown for 1–2 days before treatments were applied.

### 4.7. Complement ELISAs

Human PBMC-derived M1 macrophages and THP-1 cell line were plated and activated as described above. The next day, cells were treated with either media alone (control) or PolySia-NPs at various concentrations (0.1, 0.3, 1, and 3 mg/mL) for 24 h. Supernatant was collected 1 day post-treatment and tested immediately (1:2 sample dilution) for complement protein concentration using Quidel’s MicroVue Multiplex Complement ELISA kits (#A900, #A917; QuidelOrtho, San Diego, CA, USA) with Quansys’ Q-View imager LS (#104150GR; Quansys Biosciences, Logan, UT, USA).

### 4.8. CytoTox96 Assay

Human PBMC-derived M1 macrophages, BV-2 microglia cell line, and SHSY5Y neural cell line were plated and activated as described above. After 1–2 days, cells were treated with either media alone as a negative control (“untreated”), 10% sucrose in media as a vehicle control (“vehicle”), or PolySia-NP at various concentrations (0.01–3.0 mg/mL) for 16–24 h; or lactate dehydrogenase (LDH; max toxicity) or 10% detergent as positive controls for 30 min. Supernatants were then collected and tested immediately according to the manufacturer’s instructions (50 μL/sample, 30 min incubation) and read using a BioTek Synergy HTX plate spectrophotometer (Agilent) at 490 nm absorbance. Percent Toxicity was calculated by first subtracting the background (average optical density of wells without cells) from all experimental wells and then normalizing to 100% (maximum) toxicity (average optical density of the LDH positive control wells).

### 4.9. Mouse In Vivo Laser CNV Model

This experiment was performed by contract research organization (CRO) Powered Research (Durham, NC, USA). The mouse laser-induced choroidal neovascularization (CNV) model was used to recruit subretinal blood vessel growth from the choroid by perforating Bruch’s membrane using a diode laser. Ocular examinations were performed to confirm eye health prior to study initiation. Mice received analgesia (buprenorphine, 0.01–0.05 mg/kg, subcutaneous (SQ)), dilation (1% tropicamide HCl, 2.5% phenylephrine HCl, 0.5% proparacaine, topical), and analgesia (80–90 mg/kg ketamine, 10–20 mg/kg xylazine). PolySia-NP (low dose or high dose) or vehicle control (10% sucrose) were injected intravitreally (IVT; 1 μL) immediately before laser injury. For PolySia-NP, the low dose was 3 mg/mL, which is 0.003 mg/eye in mice (3 mg/mL × 1 μL) and approximately equivalent to 0.35 mg/eye in humans (0.003 mg/eye × 118 scaling factor) [51]; the high dose was 19.2 mg/mL, which is 0.0192 mg/eye in mice (19.2 mg/mL × 1 μL) and approximately equivalent to 2.26 mg/eye in humans (0.0192 mg/eye × 118 scaling factor) [51]. For laser CNV induction (OU), a 532 nm diode laser delivered though a split-lamp was used to create 4 spots surrounding the optic nerve. Eight days after laser CNV, mice were sacrificed via transcardial saline perfusion. Then, their eyes were enucleated and immediately fixed in 4% paraformaldehyde in phosphate-buffered saline (PBS) then stored overnight at 4 °C. The following day, the eyes were transferred to cold immunohistochemistry (IHC) buffer (PBS with 0.5% BSA and 0.2% Tween 20) until processing. Under a dissecting microscope, extraneous tissue, the anterior segment, the lens, and the retina were removed, generating eye cups.

### 4.10. Immunohistochemistry in Laser CNV Tissues

This experiment was performed by contract research organization (CRO) Powered Research (Durham, NC, USA), with additional immunohistochemical image analyses performed in house. Choroidal flat-mounts were labeled with Isolectin-B4 to assess the extent of neovascularization after laser CNV and against Iba-1, CD68, and complement C5b-9 to assess the choroidal microglia/macrophage inflammatory response to laser CNV. Eye cups were rinsed with cold IHC buffer, then incubated overnight at 4 °C with primary antibody cocktail in IHC buffer: rabbit antibody to mouse C5b-9 (Abcam; 1:100), goat antibody to mouse Iba-1 (Novus Biologicals, Centennial, CO, USA; 1:100), and/or rat antibody to mouse CD68 (Bio-Rad Laboratories, Hercules, CA, USA; 1:200). Following IHC buffer rinses, eye cups were incubated for 4 h at 4 °C with secondary antibody cocktail in ICC buffer: Isolectin-B4 DyLight 649 (Vector Laboratories, Newark, CA, USA; 1:200), donkey anti-rabbit Cy3 (Vector Laboratories; 1:200), donkey anti-goat Cy2 (Vector Laboratories; 1:200), and/or donkey anti-rat DyLight 405 (Vector Laboratories; 1:200). Following final IHC buffer rinses, the sclera–choroid/retinal pigment epithelium (RPE) complexes were then flat-mounted, covered, and sealed. Widefield fluorescent microscopy images were acquired using an Olympus Bx63 upright fluorescent microscope with CellSens (Olympus, Tokyo, Japan) software (4× and 20× images of 16–20 lesion sites per group). Analysis of neoformed vessel presence by Isolectin-B4 area (μm^2^) was also performed using CellSens software (v.4.2). Analysis of Iba1, CD68, and C5b-9 by IHC+ area (% total area) was performed in Fiji (NIH, Bethesda, MD, USA), blind to experimental group. 

### 4.11. Graphs and Statistics

All graphs and statistical analysis were generated using GraphPad prism software (v.9). Groups were compared using *t*-tests, one-way ANOVA with Dunnet post-test (versus control), or two-way ANOVA, as experimentally appropriate. Error bars are mean ± SEM. *p*-values are shown on graphs with asterisks: * *p* < 0.05, ** *p* < 0.01, *** *p* < 0.001, and **** *p* < 0.0001.

## 5. Conclusions

In conclusion, by selectively attenuating the alternative complement pathway through the sialic acid actuation of CFH-dependent decay-dissociation of amplification C3 convertase, we can regulate the complement system without disabling it. The physiologic attenuation of the complement system should demonstrate equal efficacy without the clinically realized side effects of profoundly disabling the complement system in the eye. Currently PolySia-NP, which we also found to inhibit macrophage-mediated inflammation and cytokine release, has entered human clinical trials and is being administered intravitreally in patients with geographic atrophy secondary to age-related macular degeneration. We predict that the theoretical benefits of sialic acid SAMP attenuation of the alternative complement will prove clinically beneficial with reduced adverse events.

## Data Availability

The original contributions presented in the study are included in the article/Supplementary Material; further inquiries can be directed to the corresponding authors.

## Supplementary Materials

The following supporting information can be downloaded at: https://www.mdpi.com/article/10.3390/ph17040517/s1, Figure S1: Complement multiplex ELISA on THP-1 human macrophage cell line culture supernatant.

## References

[1] Ricklin D., Reis E.S., Lambris J.D. (2016). Complement in disease: A defense system turning offensive. Nat. Rev. Nephrol..

[2] Mastellos D.C., Ricklin D., Lambris J.D. (2019). Clinical promise of next-generation complement therapeutics. Nat. Rev. Drug Discov..

[3] Mohebnasab M., Eriksson O., Persson B., Sandholm K., Mohlin C., Huber-Lang M., Keating B.J., Ekdahl K.N., Nilsson B. (2019). Current and Future Approaches for Monitoring Responses to Anti-complement Therapeutics. Front. Immunol..

[4] Portilla D., Xavier S. (2021). Role of intracellular complement activation in kidney fibrosis. Br. J. Pharmacol..

[5] Carpanini S.M., Torvell M., Morgan B.P. (2019). Therapeutic Inhibition of the Complement System in Diseases of the Central Nervous System. Front. Immunol..

[6] Clarke A.R., Christophe B.R., Khahera A., Sim J.L., Connolly E.S. (2019). Therapeutic Modulation of the Complement Cascade in Stroke. Front. Immunol..

[7] Park D.H., Connor K.M., Lambris J.D. (2019). The Challenges and Promise of Complement Therapeutics for Ocular Diseases. Front. Immunol..

[8] Sun Y., Wirta D., Murahashi W., Mathur V., Sankaranarayanan S., Taylor L.K., Yednock T., Fong D.S., Goldberg J.L. (2023). Safety and Target Engagement of Complement C1q Inhibitor ANX007 in Neurodegenerative Eye Disease: Results from Phase I Studies in Glaucoma. Ophthalmol. Sci..

[9] Hajishengallis G., Kajikawa T., Hajishengallis E., Maekawa T., Reis E.S., Mastellos D.C., Yancopoulou D., Hasturk H., Lambris J.D. (2019). Complement-Dependent Mechanisms and Interventions in Periodontal Disease. Front. Immunol..

[10] Wong W.L., Su X., Li X., Cheung C.M., Klein R., Cheng C.Y., Wong T.Y. (2014). Global prevalence of age-related macular degeneration and disease burden projection for 2020 and 2040: A systematic review and meta-analysis. Lancet Glob. Health.

[11] Heurich M., Martinez-Barricarte R., Francis N.J., Roberts D.L., Rodriguez de Cordoba S., Morgan B.P., Harris C.L. (2011). Common polymorphisms in C3, factor B, and factor H collaborate to determine systemic complement activity and disease risk. Proc. Natl. Acad. Sci. USA.

[12] Emilsson V., Gudmundsson E.F., Jonmundsson T., Jonsson B.G., Twarog M., Gudmundsdottir V., Li Z., Finkel N., Poor S., Liu X. (2022). A proteogenomic signature of age-related macular degeneration in blood. Nat. Commun..

[13] Thakkinstian A., Han P., McEvoy M., Smith W., Hoh J., Magnusson K., Zhang K., Attia J. (2006). Systematic review and meta-analysis of the association between complement factor H Y402H polymorphisms and age-related macular degeneration. Hum. Mol. Genet..

[14] Ram S., Sharma A.K., Simpson S.D., Gulati S., McQuillen D.P., Pangburn M.K., Rice P.A. (1998). A novel sialic acid binding site on factor H mediates serum resistance of sialylated Neisseria gonorrhoeae. J. Exp. Med..

[15] Schmidt C.Q., Herbert A.P., Hocking H.G., Uhrin D., Barlow P.N. (2008). Translational mini-review series on complement factor H: Structural and functional correlations for factor H. Clin. Exp. Immunol..

[16] Ranganathan S., Male D.A., Ormsby R.J., Giannakis E., Gordon D.L. Pinpointing the putative heparin/sialic acid-binding residues in the ‘sushi’ domain 7 of factor H: A molecular modeling study. Proceedings of the Pacific Symposium on Biocumputing 2000.

[17] Tolentino M., Tolentino A.J., Tolentino E.M., Krishnan A., Genead M.A. (2023). Sialic Acid Mimetic Microglial Sialic Acid-Binding Immunoglobulin-like Lectin Agonism: Potential to Restore Retinal Homeostasis and Regain Visual Function in Age-Related Macular Degeneration. Pharmaceuticals.

[18] Khanani A.M., Patel S.S., Staurenghi G., Tadayoni R., Danzig C.J., Eichenbaum D.A., Hsu J., Wykoff C.C., Heier J.S., Lally D.R. (2023). Efficacy and safety of avacincaptad pegol in patients with geographic atrophy (GATHER2): 12-month results from a randomised, double-masked, phase 3 trial. Lancet.

[19] Heier J.S., Lad E.M., Holz F.G., Rosenfeld P.J., Guymer R.H., Boyer D., Grossi F., Baumal C.R., Korobelnik J.F., Slakter J.S. (2023). Pegcetacoplan for the treatment of geographic atrophy secondary to age-related macular degeneration (OAKS and DERBY): Two multicentre, randomised, double-masked, sham-controlled, phase 3 trials. Lancet.

[20] Cruz-Pimentel M., Wu L. (2023). Complement Inhibitors for Advanced Dry Age-Related Macular Degeneration (Geographic Atrophy): Some Light at the End of the Tunnel?. J. Clin. Med..

[21] Apellis (2023). FDA Approves SYFOVRE™ (Pegcetacoplan injection) as the First and Only Treatment for Geographic Atrophy (GA), a Leading Cause of Blindness. https://investors.apellis.com/news-releases/news-release-details/fda-approves-syfovretm-pegcetacoplan-injection-first-and-only.

[22] Spaide R.F., Vavvas D.G. (2023). Complement Inhibition for Geographic Atrophy: Review of Salient Functional Outcomes and Perspective. Retina.

[23] Poor S.H., Qiu Y., Fassbender E.S., Shen S., Woolfenden A., Delpero A., Kim Y., Buchanan N., Gebuhr T.C., Hanks S.M. (2014). Reliability of the mouse model of choroidal neovascularization induced by laser photocoagulation. Investig. Ophthalmol. Vis. Sci..

[24] Gadjeva M.G., Rouseva M.M., Zlatarova A.S., Reid K.B., Kishore U., Kojouharova M.S. (2008). Interaction of human C1q with IgG and IgM: Revisited. Biochemistry.

[25] McGrath F.D., Brouwer M.C., Arlaud G.J., Daha M.R., Hack C.E., Roos A. (2006). Evidence that complement protein C1q interacts with C-reactive protein through its globular head region. J. Immunol..

[26] Nauta A.J., Daha M.R., van Kooten C., Roos A. (2003). Recognition and clearance of apoptotic cells: A role for complement and pentraxins. Trends Immunol..

[27] Roumenina L.T., Ruseva M.M., Zlatarova A., Ghai R., Kolev M., Olova N., Gadjeva M., Agrawal A., Bottazzi B., Mantovani A. (2006). Interaction of C1q with IgG1, C-reactive protein and pentraxin 3: Mutational studies using recombinant globular head modules of human C1q A, B, and C chains. Biochemistry.

[28] Bexborn F., Andersson P.O., Chen H., Nilsson B., Ekdahl K.N. (2008). The tick-over theory revisited: Formation and regulation of the soluble alternative complement C3 convertase (C_3_(H_2_O)Bb). Mol. Immunol..

[29] Bai L., Xie Q., Xia M., Gong K., Wang N., Chen Y., Zhao M. (2021). The importance of sialic acid, pH and ion concentration on the interaction of uromodulin and complement factor H. J. Cell Mol. Med..

[30] Blaum B.S., Hannan J.P., Herbert A.P., Kavanagh D., Uhrin D., Stehle T. (2015). Structural basis for sialic acid-mediated self-recognition by complement factor H. Nat. Chem. Biol..

[31] Schmidt C.Q., Hipgrave Ederveen A.L., Harder M.J., Wuhrer M., Stehle T., Blaum B.S. (2018). Biophysical analysis of sialic acid recognition by the complement regulator Factor H. Glycobiology.

[32] Varki A. (2011). Since there are PAMPs and DAMPs, there must be SAMPs? Glycan "self-associated molecular patterns" dampen innate immunity, but pathogens can mimic them. Glycobiology.

[33] Krishnan A., Sendra V.G., Patel D., Lad A., Greene M.K., Smyth P., Gallaher S.A., Herron U.M., Scott C.J., Genead M. (2023). PolySialic acid-nanoparticles inhibit macrophage mediated inflammation through Siglec agonism: A potential treatment for age related macular degeneration. Front. Immunol..

[34] Fearon D.T. (1978). Regulation by membrane sialic acid of beta1H-dependent decay-dissociation of amplification C3 convertase of the alternative complement pathway. Proc. Natl. Acad. Sci. USA.

[35] Meri S., Pangburn M.K. (1990). Discrimination between activators and nonactivators of the alternative pathway of complement: Regulation via a sialic acid/polyanion binding site on factor H. Proc. Natl. Acad. Sci. USA.

[36] Hyvarinen S., Meri S., Jokiranta T.S. (2016). Disturbed sialic acid recognition on endothelial cells and platelets in complement attack causes atypical hemolytic uremic syndrome. Blood.

[37] Biggs R.M., Makou E., Lauder S., Herbert A.P., Barlow P.N., Katti S.K. (2022). A Novel Full-Length Recombinant Human Complement Factor H (CFH; GEM103) for the Treatment of Age-Related Macular Degeneration Shows Similar In Vitro Functional Activity to Native CFH. Curr. Eye Res..

[38] Karlstetter M., Kopatz J., Aslanidis A., Shahraz A., Caramoy A., Linnartz-Gerlach B., Lin Y., Luckoff A., Fauser S., Duker K. (2017). Polysialic acid blocks mononuclear phagocyte reactivity, inhibits complement activation, and protects from vascular damage in the retina. EMBO Mol. Med..

[39] Lubbers R., van Essen M.F., van Kooten C., Trouw L.A. (2017). Production of complement components by cells of the immune system. Clin. Exp. Immunol..

[40] Hartung H.P., Hadding U. (1983). Complement components in relation to macrophage function. Agents Actions.

[41] Meri S., Pangburn M.K. (1994). Regulation of alternative pathway complement activation by glycosaminoglycans: Specificity of the polyanion binding site on factor H. Biochem. Biophys. Res. Commun..

[42] Tzoumas N., Riding G., Williams M.A., Steel D.H. (2023). Complement inhibitors for age-related macular degeneration. Cochrane Database Syst. Rev..

[43] Kim S.J., Kim J., Lee J., Cho S.Y., Kang H.J., Kim K.Y., Jin D.K. (2013). Intravitreal human complement factor H in a rat model of laser-induced choroidal neovascularisation. Br. J. Ophthalmol..

[44] Khanani A.M., Maturi R.K., Bagheri N., Bakall B., Boyer D.S., Couvillion S.S., Dhoot D.S., Holekamp N.M., Jamal K.N., Marcus D.M. (2022). A Phase I, Single Ascending Dose Study of GEM103 (Recombinant Human Complement Factor H) in Patients with Geographic Atrophy. Ophthalmol. Sci..

[45] Liao D.S., Grossi F.V., El Mehdi D., Gerber M.R., Brown D.M., Heier J.S., Wykoff C.C., Singerman L.J., Abraham P., Grassmann F. (2020). Complement C3 Inhibitor Pegcetacoplan for Geographic Atrophy Secondary to Age-Related Macular Degeneration: A Randomized Phase 2 Trial. Ophthalmology.

[46] Aviceda Therapeutics Inc. (2023). A Multiple Dose Study of AVD-104 for Geographic Atrophy (GA) Secondary to Age-Related Macular Degeneration (AMD) (SIGLEC). https://clinicaltrials.gov/study/NCT05839041.

[47] Hoh Kam J., Lenassi E., Malik T.H., Pickering M.C., Jeffery G. (2013). Complement component C3 plays a critical role in protecting the aging retina in a murine model of age-related macular degeneration. Am. J. Pathol..

[48] Tolentino M.J., Tolentino A.J. (2022). Investigational drugs in clinical trials for macular degeneration. Expert. Opin. Investig. Drugs.

[49] Shaughnessy J., Gulati S., Agarwal S., Unemo M., Ohnishi M., Su X.H., Monks B.G., Visintin A., Madico G., Lewis L.A. (2016). A Novel Factor H-Fc Chimeric Immunotherapeutic Molecule against Neisseria gonorrhoeae. J. Immunol..

[50] Shaughnessy J., Lewis L.A., Zheng B., Carr C., Bass I., Gulati S., DeOliveira R.B., Gose S., Reed G.W., Botto M. (2018). Human Factor H Domains 6 and 7 Fused to IgG1 Fc Are Immunotherapeutic against Neisseria gonorrhoeae. J. Immunol..

[51] Schmitt M., Hippelainen E., Ravina M., Arango-Gonzalez B., Antopolsky M., Vellonen K.S., Airaksinen A.J., Urtti A. (2019). Intravitreal Pharmacokinetics in Mice: SPECT/CT Imaging and Scaling to Rabbits and Humans. Mol. Pharm..

