# Supplementary material for: PolySialic Acid Nanoparticles Actuate Complement-Factor-H-Mediated Inhibition of the Alternative Complement Pathway: A Safer Potential Therapy for Age-Related Macular Degeneration
Source: Pharmaceuticals (Basel). 2024 Apr 17;17(4):517. doi: 10.3390/ph17040517 (PMC11053938; doi:10.3390/ph17040517)
Supplement: Supplementary file 1 [file pharmaceuticals-17-00517-s001.zip › pharmaceuticals-2928290-supplementary.pdf]

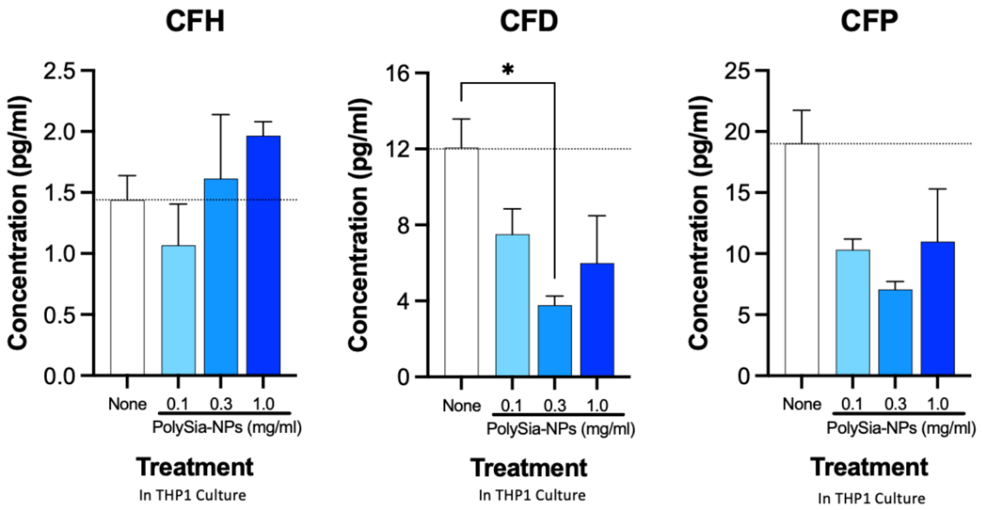

Supplemental Figure S1: Complement multiplex ELISA on THP-1 human macrophage cell line culture supernatant. * $p < 0.05$
